# The impact of the first phase of the COVID-19 pandemic on referral patterns and therapeutic service provision for children and young people’s psychosocial distress in a Low-or Middle-Income Country: A service evaluation of routinely collected data from a non-government organisation operating in schools in the Western Cape, South Africa

**DOI:** 10.1177/13591045241264861

**Published:** 2024-07-19

**Authors:** Emma Wilson, Maria E Loades, Suzanne Human, Bronwyne Coetzee, Hermine Gericke, Gerrit Laning

**Affiliations:** 1 Department of Psychology, 1555 University of Bath, UK; 2 Department of Psychology, Stellenbosch University, SA; 3 Community Keepers, SA

**Keywords:** children and young people, depression, anxiety, school-based, prevention, South Africa, LMIC, COVID-19, third sector provision

## Abstract

**Introduction:**

In low- and middle-income countries (LMICs), including South Africa, there is a paucity of psychosocial support services. Therefore, services are often provided in schools by non-government organisations like Community Keepers (CK). The COVID-19 pandemic and resultant restrictions meant that children and young people’s (CYP) lives changed, negatively affecting their mental health. Further, organisations like CK had to change their working processes.

**Method:**

This project compared routinely collected data from CK from 2019 (pre-pandemic) to 2020 (pandemic) to describe the changes that occurred in referral patterns to, and service provision by, CK.

**Results:**

Both pre-pandemic and during the pandemic, most referrals of CYP were for emotional/psychological support and behavioural difficulties. In 2020, referrals for general guidance increased, whilst referrals for peer group issues and sexuality decreased. Further, CK completed more brief check-ins, provided wellbeing workshops to increased numbers of teachers, parents and CYP, and had more consultation sessions with other service providers during the pandemic.

**Discussion:**

Routinely collected data from this community-based service in a LMIC context shows differences in the way that support was provided, and to whom, during the COVID-19 pandemic. Clinical implications, including the importance of increasing access to psychosocial support via technology, are included.

## Plain language summary

The COVID-19 pandemic disproportionately impacted children, young people and families who are most vulnerable, including those in low- and middle-income countries (LMICs). Within LMICs, the lack of trained psychological professionals and investment in mental healthcare interventions means access to help is limited. School-based provision, which may increase access to mental health support was curtailed during school closures when face-to face service provision was prohibited. It is well-documented that the COVID-19 pandemic impacted CYP’s mental and emotional well-being. However, it is less well understood how the need and service provision of third sector non-government organisations changed during this time. Thus, we partnered with a non-government organisation, Community Keepers (CK) who are based in Western Cape, South Africa to understand how referral patterns changed from 2019 (pre-pandemic) to 2020 (peri-pandemic) and explore the specific adaptations that CK made to service provision. CK aims to provide free mental health care services to CYP, their parents (legal guardians / primary caregivers; henceforth referred to as parents) and teachers, on-site at schools (www.communitykeepers.org). Results suggest that both pre-pandemic and during the pandemic, most referrals of CYP were for emotional/psychological support and behavioural difficulties. In 2020, referrals for general guidance and chronic/serious illness increased, whilst referrals for peer group issues and sexuality decreased. Further, CK completed wellbeing workshops to increased numbers of teachers, parents and CYP, and had more consultation sessions with other service providers during the pandemic. In addition, COVID-19 restrictions necessitated a reduction in face-to-face sessions and a move towards more systemic support as well as telephonic and/or online support to individuals. This paper demonstrates that should another global crisis like the Covid-19 pandemic occur, necessitating school closures and/or restrictions to in-person interaction, it will be important for organisations working in schools, particularly with CYP who are particularly vulnerable, to pivot their provision to enable continued support. Further, specific recommendations for CK were given regarding data collection, that can be used to support growth and understanding service provision patterns across similar settings.

Please provide up to three (3) brief statements outlining what is already known about this topic (no more than 25 words per statement):

(1) The COVID-19 pandemic disproportionately impacted children, young people and families who are most vulnerable, including those in low- and middle-income countries (LMICs).
(2) Within LMICs, the lack of trained psychological professionals and investment in mental health care interventions means access to help is limited.
(3) While school-based provision may increase access to mental health support, this was curtailed during school closures when face-to face service provision was prohibited.

Please provide up to three (3) brief statements detailing what this study adds to the topic (no more than 25 words per statement):

(1) By comparing 2020 to 2019 data from a school-based counselling organisation, we found that the pattern of reasons for referral changed significantly during the pandemic.
(2) Staff members were required to do more work navigating the impact of inequality in social determinants during 2020, highlighting some of the socio-economic challenges that occurred.
(3) COVID-19 restrictions necessitated a reduction in face-to-face sessions and a move towards more systemic support as well as telephonic and/or online support to individuals.

## Introduction

**Figure 1.**
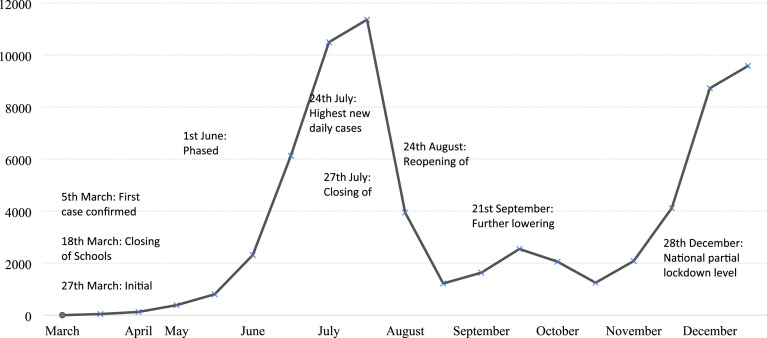
Daily new cases of COVID-19 in South Africa with significant events and progression (Coetzee et al., 2022; WHO, 2020.

Children and young people (CYP) in low-and-middle-income countries (LMICs), including South Africa, are disproportionately vulnerable to developing mental health problems due to being exposed to multiple risk factors such as violence, maltreatment, growing up in households affected by HIV/AIDS, and poverty (Flisher et al., 2012; Kieling et al., 2011; Lu, Li, & Patel, 2018; Patel et al., 2007). In response to the COVID-19 pandemic, the South African government implemented various disease containment measures such as school closures, maintaining of physical distance between people, and the wearing of face masks (see Figure 1 for a timeline of events) (Coetzee et al., 2022; World Health Organisation, 2020). The COVID-19 pandemic and its sequelae impacted CYP’s mental health (Nearchou et al., 2020; Slomski, 2021; Kauhanen et al., 2022) and also constrained psychosocial support services which had traditionally operated face-to-face in communities. In this paper, we report on how the first phase of disease containment measures, particularly school closures, affected service delivery for Community Keepers (CK), a community-based (primarily school-based) South African non-government organisation (NGO).

There is growing evidence globally regarding the impact the pandemic and resultant restrictions had for CYP mental health, although less is known about the impact in a LMIC context, and South Africa specifically. Whilst some CYP reported positive experiences during the COVID-19 pandemic (Demkowicz et al., 2021), for many, the pandemic negatively impacted their psychosocial functioning and resulted in increased levels of depression and anxiety (Nearchou et al., 2020; Slomski, 2021; Kauhanen et al., 2022; Dworzanowski-Venter, 2021). In the Western Cape in South Africa, qualitative data obtained through individual telephonic interviews with CYP (aged 12–13 years), their parents, teachers, and CK staff members, found that CYP experienced frustration, anxiety, and loneliness during school closures (Coetzee et al., 2022).

Psychosocial distress and mental health problems have negative consequences in both the short and long-term, yet there is typically a gap between needs and access to psychosocial support in LMICs. For example, adolescent depression is associated with failure to complete secondary school and unemployment (Finning et al., 2019). To mitigate this, it is vital that CYP receive timely support if they are experiencing mental health difficulties however, in South Africa, there is a huge gap (∼90%) between the mental health needs of CYP and the availability of resources as only half of public hospitals that offer mental health support have a resident psychiatrist, and one third do not have a clinical psychologist (Daniels, 2021; Docrat et al., 2019).

Given the dearth of other mental health care provision, support provided in schools by NGOs is key to meeting the need for psychosocial support for CYP particularly as in South Africa, 98% of children of school-going age (7–17 years) attended some form of educational facility in 2018 (Hall, 2022). Community Keepers (CK), a South African non-government organisation (NGO), which was established in 2008 aims to provide free mental health care services to CYP, their parents (legal guardians/primary caregivers; henceforth referred to as parents) and teachers, on-site at schools. CK receives funding from state and privately-owned-enterprises, as well as individuals, and because they are located within school premises, CYP are able to readily seek and access mental health support. CK’s multifaceted approach provides different forms of support to CYP and their networks (see Table 1), however, for much of 2020, CK’s usual model of face-to-face service provision was disrupted considerably by the restrictions that were imposed to contain the COVID-19 pandemic.

**Table 1.** Definitions of CK’s Subcategories of Service Provision.

Subcategory of service provision	Definition
Support keepers	Individual and small group psychosocial therapeutic counselling provided to CYP (mostly) and teachers
Life keepers	Workshops provided to CYP with the aim of developing their social and personal skills and to build resilience
Parent and teacher keepers	Workshops for parents and teachers respectively, aimed at equipping them with the knowledge and skills to support CYP.
Connect keepers	CK staff members who connect and build relationships with other service providers
Learner keepers	Committees made up of CYP who serve as advisers and champions at each school

Whilst we have some understanding of how COVID-19 and disease containment measures influenced CYP’s mental health and wellbeing, we know little about how community-based mental health care service providers, such as CK, which operated within schools, adapted to continue to provide their services during the pandemic when children were not in attendance.

Therefore, using CK’s routinely collected data, we aimed to understand how referral patterns changed from 2019 (pre-pandemic) to 2020 (peri-pandemic) and explore the specific adaptations that CK made to service provision. The specific questions we sought to answer were:

(1) What changes (if any) were there in the:
  (a) number of referrals of CYP to CK;
  (b) pattern of referral reason of CYP to CK;
  (c) pattern of referral source of CYP to CK, in other words, who referred CYP to CK; and
  (d) pattern of demographics of referred service users?
(2) Were there differences in the:
  (a) proportion of referred CYP who were offered therapeutic input;
  (b) demographics of individuals (e.g. age, gender) who received input;
  (c) therapeutic input offered (e.g. number of sessions, type of therapeutic input);
  (d) target of the intervention (e.g. individual, family, school, ecosystem); and
  (e) medium of care provision (e.g. in-person, telephonic, online)?

## Method

### Study design

We conducted a quantitative analysis of existing, anonymised data routinely collected by CK.

### Research setting

CK provides a range of support to CYP, their parents and teachers (see Table 1). CK views their mission as investing in the social and emotional wellbeing of CYP and to help create supportive school communities where learning and development can prosper (https://www.communitykeepers.org).

At the end of 2020, CK had offices within 28 schools, situated in 14 different municipal areas in the Western Cape Province of South Africa (Statistics of South Africa, 2011). Many of these schools are eligible for funding from South Africa’s National School Nutrition Programme (Devereux et al., 2018) because many of the learners come from low socio-economic status (SES) families. We used data collected from the South African national census of 2011 (Statistics South Africa, 2011) to describe the areas where the schools are situated (see Table 2).

**Table 2.** Demographic Information (2011 National Census) on the 14 Areas in Which CK Operated in 2020, Compared to Provincial (Western Cape) and National (South Africa) Averages.

Demographic variables	The 14 areas in which CK operated	Provincial averages	National averages
Population group^a^ (%, range)			
White	29.4 (0.2–82.2)	15.7	8.9
Black African	15.3 (3.3–94.6)	32.9	79.2
Coloured	39.8 (4.7–93.9)	48.8	8.9
Indian/Asian	0.6 (0–3.4)	1.0	2.5
Other	2.4 (0–5.0)	1.6	0.5
First language (%, range)			
Afrikaans	57.1 (5.9–94.6)	49.7	13.5
English	11.1 (1.9–67.7)	20.2	9.6
isiXhosa	8.1 (0–84.9)	24.7	16.0
Other	5.8 (2.0–12.4)	-	-
Household members (%, range)			
Young people (0 – 14 years of age)	23.7 (18.4–30.3)	-	29.2
Elderly (65+ years of age)	5.4 (1.3–12.3)	-	5.3
Sex^b^ (%, range)			
Male	48.3 (43.1–50.2)	-	48.7
Female	51.4 (49.5–56.9)	-	51.3
Highest level of education amongst persons aged 20 years and older (%, range)			
No schooling	2.6 (0–8.4)	2.7	8.6
Some primary school	16.2 (1.3–24.2)	10.7	12.3
Completed primary school	7.0 (0.7–9.7)	5.6	4.6
Some secondary school	40.3 (11.5–51.0)	38.3	33.9
Completed secondary school	25.5 (14.6–38.0)	28.2	28.9
Higher education	19.4 (1.1–52.4)	14.4	11.8
Household infrastructure (%, range)			
Formal dwelling	78.8 (31.2–100)	80.4	77.6
Flush toilet connected to sewage	89.4 (67.7–98.9)	91.6	90.6
Piped water inside dwelling	79.5 (33.1–98.8)	99.1	91.2
Household income (%, range)			
% Of households with no income	12.3 (0–29.4)	-	-

*Note.* We calculated weighted averages for the various demographic variables for the 14 areas in which CK operated in 2020. Not all percentages add up to 100 due to rounding. Some of the provincial and national averages aren’t reported on in this table, since the authors were unable to access those data.

^a^These classifications for population group are the classifications as used in the South African national census of 2011.

^b^These classifications for sex are the classifications as used in the South African national census of 2011. As far as the authors of this paper now, the census data did not collect any data on sex and/or gender other than male or female.

### Participants

The participants in this study were the CYP, their parents and teachers, and other service providers that CK staff connected with (see Table 1) during 2019 and 2020 (Human et al., – under review).

### Measures

#### Demographic variables

For Support Keepers work with CYP, CK staff collect demographic information about the individual including age, biological sex at birth, and school year (referred to as grade). This information is collected at the point of referral by the referrer.

#### Referral source

Referral source is coded into one of five categories - self-referral; parent (caregiver/guardian); teacher; Community Keepers (CK staff can refer a child for therapy); or outside organisation (e.g., welfare services).

#### Referral reason

The referral reason is determined by CK staff using self-reported information from the individual and the person who referred the individual.

#### Intervention focus

Upon the completion of an intervention, CK staff record the focus of the intervention (as determined by CK staff at the end of the support using the categories shown in table 7) and the number of sessions provided.

#### Session attendance and facilitation

For indirect work including Parent, Teacher, Connect, and Learner Keepers, the CK staff members recorded the number of individuals who attended each session, as well as the number of sessions that were facilitated.

#### Telephonic service provision

Regarding telephonic service provision that was provided in 2020 because of the COVID-19 pandemic only the number of minutes spent per call, and not the number of specific sessions, referral reason, or intervention focus, were recorded.

### Procedure

The routinely collected data is captured on a school specific Microsoft Excel database by a full-time Office Manager at each school in which CK is based. Each school has a unique abbreviation, and everyone who accesses support is assigned a unique number. Therefore, all data is anonymised at the point of entry onto the school specific Excel database. The data is then collated and checked across each school and provided to the CK Operations Manager, who edits the master Excel database with this information. The data used for this study were extracted from this master Excel database.

### Data analysis

Quantitative data was analysed descriptively, with inferential statistics used where appropriate. We used Chi-squared tests of association and follow up Bonferroni corrected Z-tests of difference. Where we refer to results as statistically significant this is at the *p* ≤ 0.05 level. Microsoft Excel was used to calculate percentage change and SPSS version 29.0.1.0 was used for Chi-squared and Z-test analysis.

### Ethical approval

Ethical approval for conducting descriptive studies on the community-based, therapeutic support provided by Community Keepers NGO in South Africa, both pre-pandemic (Human et al., under review) and during the pandemic (this project) was by the Research Ethics Committee: Social, Behavioural and Education Research (REC: SBER), Stellenbosch University (Project ID: 24381). Reciprocity was received from the Psychology Research Ethics Committee (PREC), University of Bath (22-035).

## Results

In 2020, Support Keepers services were provided to 6862 CYP, compared to 2769 CYP in 2019. Also including Teacher Keepers, Parent Keepers, and Connect Keepers, CK reached a combined total of 37471 individuals in 2020, compared to 34676 individuals in 2019.

### In-person support provision in 2019 and 2020

CYP Support Keeper referrals increased by 88, from 2769 (2019) to 2857 (2020). Gender of Support Keeper CYP did not change significantly between the two years, x^2^(1) = 2.557, *p* = .110, and neither did CYP’s nationality x^2^(13) = 13.122, *p* = .438 (see Table 3).

**Table 3.** The Number of Referrals of CYP to Support Keepers Categorised by Gender and Nationality in 2019 and 2020 (%).

Demographic characteristics	2019 (*n* = 2769)	2020 (*n* = 2875)	Percentage change (%) from 2019 to 2020
Sex (%)			
Male	46.2%	44.0%	−2%
Female	53.8%	56.0%	2%
Nationality (%)			
South african	95.4%	96.4%	1.0%
Zambian	0.1%	0.1%	−3.1%
DRC	2.3%	1.4%	−40.0%*
Angola	0.1%	0.1%	−27.3%
Zimbabwe	1.3%	1.2%	−10.9%
Burindi	0.1%	0.1%	29.2%
Ethiopian	0.0%	0.0%	−100.0%
Cameroon	0.1%	0.1%	−3.1%
Rwanda	0.0%	0.0%	0.0%
Botswana	0.0%	0.0%	−100.0%
Namibian	0.0%	0.0%	−3.1%
Malawian	0.2%	0.1%	−22.5%
Kenya	0.0%	0.0%	−100.0%
Other	0.2%	0.4%	93.8%

*Denotes a z test significant change at a 0.05 level, in proportion of referrals between 2019 and 2020.

**Figure 2.**
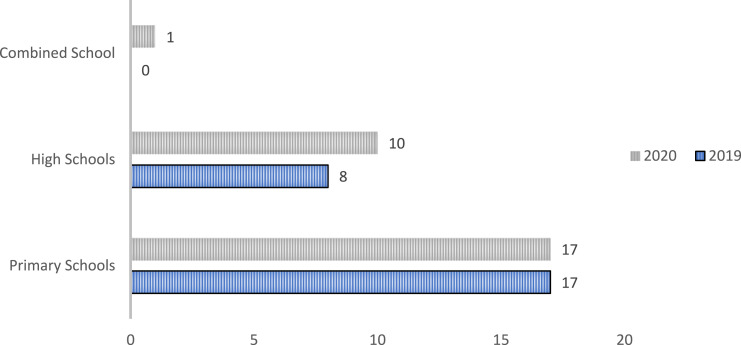
A graph depicting the number of high schools, primary schools, and combined schools CK were partnered with in 2019 and 2020.

**Figure 3.**
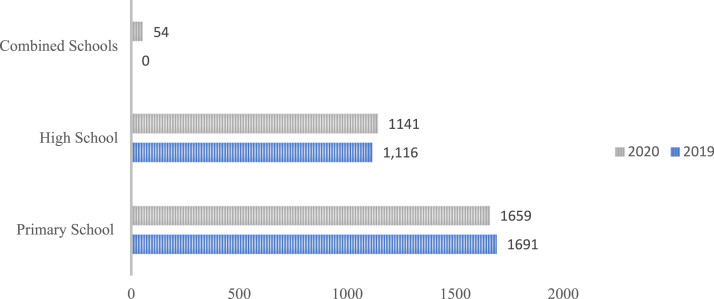
A graph depicting the number of referrals from high school-aged children, primary school-aged children, and combined schools in 2019 and 2020.

As shown in Figure 2, CK’s number of partner schools increased by 12% in 2020. The age patterns of referrals of CYP to Support Keepers remained largely the same throughout the two years (Figure 3).

### Referrals of CYP to support keepers in 2019 and 2020

**Figure 4.**
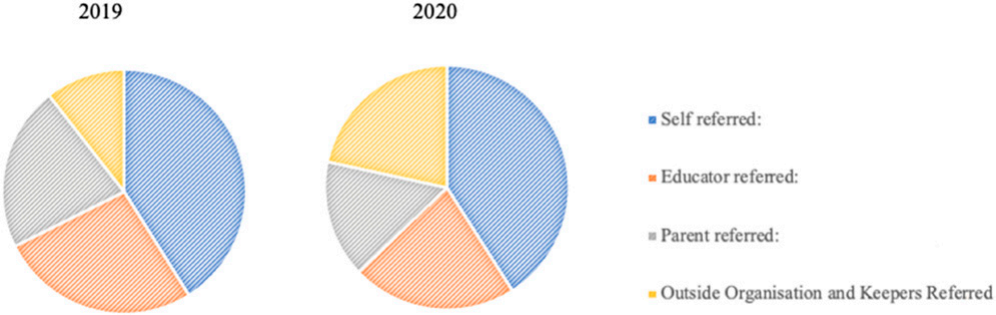
Pie chart showing referral source of CYP to Support Keepers in 2019 (left) and 2020 (right).

Table 4 The overall pattern of referral source significantly changed from 2019 to 2020; x^2^(3) = 148.655^,^ *p* = <0.001. Cross-tabulation z-tests demonstrated a significant decrease in referrals from teachers (−5%) and parent referrals (−6%) alongside an increase in outside organisation referrals (21.8%), however self-referral rates remained similar across the two years. Most CYP who made use of Support Keepers service referred themselves, both in 2019 and 2020. (see Figures 4).

**Table 4.** The Number of Referrals Categorised by Source in 2019 and 2020 (%).

*Referral Source*	2019 (*n* = 2769)	2020 (*n* = 2875)	Percentage Change 2019 to 2020
Self-referred	41.1%	41.0%	0%
Teacher-referred	26.5%	21.9%	−5%^a^
Parent-referred	21.8%	15.5%	−6%^a^
Outside organisation	10.5%	21.6%	11%^a^

^a^Denotes a proportion that does significantly differ from each other at the *p* ≤ .05 level.

In addition, the trend regarding referral reason significantly changed across 2019 and 2020; X^2^(9) = 307.685, *p* - <0.001. For both years, emotional/psychological problems and behavioural problems were the two most common reasons for referral of CYP to Support Keepers. Apart from ‘not known’, all categories saw a significant percentage change across the two years. Large increases in referrals for general guidance (1172.9%), speech (772.3%) and chronic illness (102.4%) were seen in 2020 compared to 2019; alongside decreased referrals for sexuality (−49.7%) and peer group issues (−46.8%) (see Table 5).

**Table 5.** Support Keepers for CYP: Percentages of Referral Reason in 2019 and 2020, Alongside the difference in percentage Between years. N = number of CYP’s.

Referral reason	2019 (*n* = 2769)	2020 (*n* = 2857)	Percentage change 2019 to 2020
Family/Community problems	20.9%	16.6%^a^	−20.5%^a^
Behaviour	22.4%	17.5%^a^	−21.6%^a^
Peer group	10.3%	5.5%^a^	−46.8%^a^
Sexuality	6.6%	3.3%^a^	−49.7%^a^
Emotional/Psychological problems	30.6%	37.3%^a^	22.0%^a^
Chronic/Serious illnesses	1.2%	2.5%^a^	102.4%^a^
Scholastic	3.3%	5.6%^a^	72.3%^a^
Speech	0.0%	0.3%^a^	772.3%^a^
General guidance	0.5%	6.9%^a^	1172.9%^a^
Not known	4.2%	4.4%	6.1%

^a^Denotes a proportion that does significantly differ from each other at the *p* ≤ .05 level.

NB. Please note that the category of sexuality is a term used for individuals who are having difficulty with areas relating to questioning their sexual orientation and/or gender identity and/or have questions about sexual activity.

### Service provision in 2019 and 2020

#### In-person service provision in 2019 and 2020

COVID-19 disease containment measures such as school closures meant that during 2020 there was a period when CK were not allowed to facilitate in-person service delivery at their collaborating schools. When looking specifically at Support Keepers for CYP, the total number of individual sessions offered reduced by 30.4% from 2019 to 2020 (16461 session in 2019, to 10951 sessions in 2020). The average number of sessions per service user was 2.77 in 2020, compared to 4.33 in 2019.

The overall change in trend of service provision was statistically significant between 2019 and 2020; x^2^(9) = 250.254^,^ *p* = <0.001. All categories of service provision changed significantly, apart from statutory work, social worker, and teacher sessions. Provision of check in sessions increased significantly in 2020 (a 39.8% increase) whilst provision of group sessions (both as an adjunct to individual therapy and as the primary provision) reduced significantly by −53% and −82.1% respectively (see Table 6).

**Table 6.** Service Provision to CYP as Part of Support Keepers in 2019 and 2020 (%); Alongside the Percentage Change of Service Provision Over the Two Years, Where *n* = Number of Sessions.

Session type	2019 (*n* = 16461.76)	2020 (*n* = 10951)	Percentage change from 2019 to 2020
Individual sessions with learners: 40–45 minutes contact time	67.5%	70.5%^a^	4.5%^a^
Groups as part of individual therapy: 40–45 minutes contact time	1.9%	0.9%^a^	−53.5%^a^
Parent: 1 hour contact time	8.8%	6.4%^a^	−27.8%^a^
Social worker: 1 hour contact time	1.2%	1.2%	−1.4%
Teacher: 15 minutes contact time	11.1%	11.4%	3.1%
Outside service provider: 1 hour contact time	1.4%	1.2%	−15.7%
Classroom observations: 1 hour	0.8%	0.6%^a^	−27.2%^a^
Check-in sessions: 15 minutes contact time = 0.25 recorded	4.1%	5.8%^a^	39.8%^a^
Statutory work: 45 minutes doing related work	1.7%	1.8%	9.3%
Group therapy sessions: 40–45 minutes contact time	1.5%	0.3%^a^	−82.1%^a^

^a^Denotes a proportion that does significantly differ from each other at the *p* ≤ .05 level.

In both 2019 and 2020, most Support Keepers sessions for CYP focused on emotional/psychological, family and behaviour problems. All categories within topic of intervention changed significantly across the two years apart from family/community problems. In 2020, the biggest reduction in terms of focus of psychosocial support was seen in peer group problems (−47.3%) and interventions which focused on sexuality (−41.8%). While the largest increases in focus of psychosocial support were general guidance (1318.1%), speech (530.0%), and chronic/serious illness (263.4%). These changes in focus of CYP intervention were statistically significantly, x^2^(9) = 442.217, *p* = <0.001. This trend mirrors that of reason for referral Table 7.

**Table 7.** Percentage Change in the Number of Individuals Reached and Sessions Provided as Part of Life Keepers, Parent Keepers, Teacher Keepers, Learner Keepers, and Connect Keepers From 2019 to 2020, Where *n* = Number of Individuals.

Target topic of intervention	2019 (*n* = 2769)	2020 (*n* = 2875)	Percentage change from 2019 to 2020
Family/Community problems	20.8%	20.6%	−0.9%
Behaviour	15.0%	11.8%^a^	−21.3%^a^
Peer group	8.6%	4.6%^a^	−47.3%^a^
Sexuality	5.8%	3.4%^a^	−41.8%^a^
Emotional/Psychological problems	27.5%	32.0%^a^	16.1%^a^
Chronic/Serious illnesses	0.7%	2.6%^a^	263.4%^a^
Scholastic	2.9%	5.9%^a^	104.7%^a^
Speech	0.1%	0.5%^a^	530.0%^a^
General guidance	0.7%	9.7%^a^	1318.1%^a^
Not known	17.9%	9.0%^a^	−49.8%^a^

^a^Denotes a proportion that does significantly differ from each other at the *p* ≤ .05 level. NB. When “Not known” recorded at the end of an intervention, it may be indicative of interventions that are still in process at the end of the calendar year.

Further, statistically significant changes were seen in the overall pattern of intervention provision for CYP’s networks (interventions provided as part of Parent, Teacher, Learner and Connect Keepers) x^2^(15) = 2440.966, *p* < .001. There was a statistically significant increase in the number of individuals attending workshops in small groups (27.3%), Parent (48.2%), Teacher (32.1%) and Connect Keeper sessions (36.2%), alongside the amount of people attending management committee (35.0%) meetings. The amount of Learner Keepers service users did not change significantly, despite the number of sessions offered reducing significantly (percentage reduction = 42.5%) (see Table 8). Similarly, a significant reduction was seen in the number of small group workshops (−5.8%) and Life Keepers programmes (−89.5%). An inverse trend was seen for big group sessions, with the number of sessions provided significantly increasing by 126.7%.

**Table 8.** Percentage Change in the Number of Individuals Accessing and Sessions Provided for Life Keepers, Parent Keepers, Teacher Keepers, Learner Keepers and Connect Keepers From 2019 to 2020.

Type of intervention provided by CK in 2019 and 2020	Percentage change (%) in the number of individuals reached by intervention type in 2020 from 2019	Percentage change (%) in the number of sessions provided by intervention type from 2019 – 2020
Life keepers		
Workshops facilitated with small groups of CYP (for example a sub-set of CYP in a specific grade)	27.3%^a^	−5.8%^a^
Workshops facilitated with big groups of CYP (for example an entire grade)	−21.7%^a^	126.7%^a^
Programmes offered to CYP consisting of more than one session	−89.2%^a^	−89.4%^a^
Parent keepers		
Parent sessions (psychosocial support to parents/caregivers/guardians of CYP)	48.2%^a^	19.0%
Parent committee sessions (CK staff and parents/caregivers/guardians getting together to connect and plan service delivery)	−60.0%^a^	
Teacher keepers		
Workshops on professional development	32.1%^a^	76.9%^a^
Management committee (CK staff and teachers getting together to connect and plan service delivery)	35.0%^a^	−62.9%^a^
Learner keepers		
Parent sessions (parent sessions linked to the therapeutic interventions for individual learners. The session would then revolve around capacitating the parent to support their child better)	−1.8%	−42.5%^a^
Connect keepers		
Connect sessions (CK staff and staff members from other organisations getting together to connect and plan service delivery)	36.2%^a^	

^a^Denotes a proportion that does significantly differ from each other at the *p* ≤ .05 level.

### Telephonic service provision

Between March and December 2020, 4005 additional Support Keepers referrals were made to the service for telephone support. Support via telephone was not provided in 2019 and was a response to COVID-19 restrictions. Of these referrals, 40.4% (*n* = 1,618) were of high school aged service users; 56.7% (*n* = 2,270) were referred from primary schools; and 2.9% (*n* = 117) were from a combined school. Most of these referrals were made by an Outside Organisation or internally referred within CK. Self-referred was the second most common referral route, then teacher referred. Parents were the least likely route of referral.

CYP and parents were the main targets for these interventions and together were provided with over 1000 hours of telephonic support (Table 9). The referral reason, and intervention focus was not recorded.

**Table 9.** The Session Type and Number of Minutes of Telephonic Contact in 2020.

Session type	Total minutes telephonic support provided
Individual	29808.94
Parent	30488.22
Teacher	6716.98
Social worker	1498.15
Statutory	814.09
Outside organisation	2660.62

## Discussion

Using routinely collected referral and provision data from 2019 and 2020 from a school-based psychosocial support NGO in South Africa, Community Keepers, we found that referrals of CYP for psychosocial support increased in the peri-pandemic phase of COVID-19 restrictions (2020), when compared to pre-pandemic (2019) numbers. Whilst the demographics of the CYP who were referred in 2020 were not significantly different to those 2019, patterns of referral source, referral reason, and the nature of interventions provided, all changed significantly. Large increases in referrals for general guidance, chronic/serious illness, and scholastic problems were seen in 2020, whereas referrals for support related to sexuality and peer problems decreased in comparison to 2019.

Our findings suggest an increased need for psychosocial support during 2020 which does not correlate directly with global patterns as a systematic review of 29 studies reported that many mental health services saw an initial decrease in referrals during the first lockdown period in 2020, with some reporting an increase back towards normal levels during the latter part of the year. Some then saw an increase that continued into 2021; therefore, whilst all services reported a change in referral rates with the advent of the pandemic, the direction of this change varied with the stage of the pandemic and service context (Duden et al., 2022). The increased need for psychosocial support may reflect the impact of school closures on CYP in South Africa, which limited access to existing support systems outside of the family home. It may also reflect heightened anxiety in response to the global threat of COVID-19 and the dramatic changes in daily life that disease containment measures like stay-at-home orders necessitated.

Psychosocial support in schools may be particularly important in LMIC contexts like South Africa where CYP are disproportionately vulnerable to developing mental health difficulties, given their circumstances of poverty and high levels of violence (Hiller et al., 2017). With pandemic restrictions, South Africa saw an increase in poverty and a lack of food aid (Mbunge, 2020; Stiegler & Bouchard, 2020). Within LMICs, beyond academic learning, schools are also an access point for food for CYP (Durao et al., 2020). Schools therefore provide a safe space in which the basic needs of daily living as well as psychosocial support can be accessed, and school closures disrupted this for many, with a likely impact on mental health and wellbeing.

Our findings that referrals for sexuality issues reduced contrasts with findings from other contexts. For example, a qualitative study on the impact of COVID-19 on sexual and gender minorities (SGM) conducted in USA found that young people described increasing distress in lockdown due to inability to be around supportive and accepting communities (Batra et al., 2023). However, it could be that due to social and cultural non-acceptance of homosexuality in SA, CYP with these difficulties were not able to discuss them with others, without a safe and private space to do so, particularly when much of the psychosocial support was accessed telephonically from within the family home (Gyamerah et al., 2019). Further, pre-pandemic research suggests that many SGM adolescents face bullying, violence, or discrimination (DeMulder et al., 2020; Mavhandu-Mudzusi, 2017) and thus, spending time away from non-accepting others may have reduced the need for psychological support. It is important that more research is conducted, specifically in SA and other LMICs, to understand the pattern found in this report and to address the needs of this stigmatised population.

Within CK, the category of general guidance has been used as an umbrella term by the clinicians. Under this term the work typically focuses on managing the impact of social injustices and inequalities on CYP’s well-being, for example, when CYP are suffering from being hungry, not sleeping, and/or struggling with the consequences of crime, poverty, and other negative social determinants. Thus, as there was an increase in lack of food supplies, increased unemployment, and increased need for social services during COVID-19 (Hart et al., 2022; Ingle et al., 2020), the impact on CYP children was likely reflected in CK’s work. This insight from CK underscores the importance of understanding the socio-economic contexts of the communities in which service delivery takes place, so that service providers can plan and/or adapt their services accordingly.

This increased need for general guidance was seen globally, as was evidenced by the World Health Organization (WHO) releasing recommendations to deliver a conceptual framework for mental health during the COVID-19 pandemic within the first few months of the outbreak (Ransing et al., 2020). One of the recommendations was for mental health service providers to train mental health care professionals to provide and disseminate what they referred to as Psychological First Aid (PFA) (Ransing et al., 2020). PFA includes delivering information regarding self and family care, nutrition, sleep, rest, exercise; relaxation and coping strategies; facilitating connectedness; fostering hope; and giving information regarding identification of red flags or warning signs (Ransing et al., 2020). The introduction of brief, telephonic check-in sessions that CK staff members had with individuals during 2020 could reflect this notion of PFA. This pattern also explains the increase in check-in sessions provided by CK.

Given the limited access to psychosocial support, it is important for CK, and other NGOs to consider how they can maximise these consultation and check-in sessions to expand the interventions they can provide and increase accessibility.

As CK’s original mode of service delivery was to deliver psychosocial services in-person on school premises, they needed to adapt and shift their practice dramatically when schools were closed. To adhere to disease containment measures, such as maintaining physical distance among individuals, CK started to reduce their group sessions and increased their individual and one-to-one sessions, which explains the significant change regarding intervention type. CK successfully completed over 1000 hours of telephonic psychosocial support for individuals and the supporting systems of people around them.

Whilst the COVID-19 crisis has fast-forwarded the use of technology in mental health care generally, in LMICs, not all individuals have equal access to technology and in South Africa mobile data services are costly (Phokeer, Johnson, & Feamster, 2016) which limited the efficacy of this trend. CK thus responded by having staff members call individuals, so that the telephone calls did not cost the client’s money in terms of airtime. NGOs and statutory service provision should ensure that this is continued to reduce a barrier faced by individuals from low socio-economic status households and/or communities.

### Strengths and limitations

A strength of this study is the large sample using routinely collected data and including patterns of referrals and service provision over two 12-month periods**.** This report also goes someway to increase the voices of, and identity the issues faced, by those often unheard due to socio-economic disadvantage. However, the following limitations should be noted. Firstly, we have not been able to give fine grain detail to the timeline of the changes in patterns of referrals and service interventions, such as correlating the specific months of tighter restrictions, or moments when schools re-opened. In addition, we have not included data from 2021 and beyond, and further work could explore how changes were reported in that period to inform activity from the initial pandemic period into the post-pandemic era. Furthermore, due to the data collection methods and data that were available, we could not report on whether there was an increase or decrease in referrals that were not accepted onto the CK caseload. Lastly, future studies should also investigate changes in outcomes on standardised measures.

### Clinical and research implications

Should another global crisis like the COVID-19 pandemic occur, necessitating school closures and/or restrictions to in-person interaction, it will be important for organisations working in schools, particularly with CYP who are particularly vulnerable, to pivot their provision to enable continued support. Our study has demonstrated how this was done in 2020 in one organisation, for example, by increasing telephonic support and by also taking on more of a co-ordination role with other services in the absence of schools to support this. This pivot in services demonstrates how organisations like CK rose to the extreme challenges posed to us all, within their context and their specific restrictions.

More broadly, whilst it is helpful to leverage routinely collected data to capture unmet need, it may be helpful for organisations providing psychosocial support in community settings to routinely record the total number of referrals into the service and appropriateness of referral in addition to recording data once the referral is accepted. It is also key that organisations find ways to record remotely provided support (e.g., telephone sessions in this instance) in a way that is comparable to face-to-face service provision. This record will both recognise the value of this provision as an activity and allow for more detailed comparisons to be made between the two provision types.

Future studies should track the needs of CYP coming out of the pandemic and returning to schools, for example, it would be interesting to know whether there are now more CYP being referred for peer related issues or requiring support for their sexuality. It will also be important to examine the helpfulness and utility of telephone support to continue to provide psychosocial support that is school-based during periods of school closures, whilst also considering how this mode of support may be helpful for some individuals even whilst schools are open. Alongside this, it is useful to consider the impact of providing telephonic services in light of the unequal access to technology in LMICs and whether this increases accessibility and/or increases a barrier for some.

## Conclusion

As a result of the COVID-19 pandemic, CYP have faced adversities, school closures and uncertainty. Therefore, CYP in LMICs such as South Africa faced additional challenges on top of systemic difficulties such as relative poverty, poor health and increased likelihood of trauma. This was compounded by their regular source of support being unavailable to them due to school closures and restrictions. Thus, routinely collected data on referrals and provision from one NGO in South Africa suggests that the need for psychosocial support for CYP significantly increased during the pandemic year, 2020 and there was an urgent need for the service provider, CK, to find new ways to deliver support. The increased need was predominantly for emotional/psychological support, support for behaviour and general guidance. Aligned with the nature of the restrictions on in-person social interactions, the services provided by CK shifted towards more telephonic support and away from large group interventions. This research highlights the need to understand how services providing psychosocial support in community contexts in LMICs can pivot in response to changing circumstances.
